# Supplementary material for: Brain responses to different types of salience in antipsychotic naïve first episode psychosis: An fMRI study
Source: Transl Psychiatry. 2018 Sep 21;8:196. doi: 10.1038/s41398-018-0250-3 (PMC6154975; doi:10.1038/s41398-018-0250-3)
Supplement: Supplementary file 1 — Supplementary material [file 41398_2018_250_MOESM1_ESM.docx]

**Supplementary material**

**Brain responses to different types of salience in antipsychotic naïve first episode psychosis: An fMRI study**

Franziska Knolle^1,3^*, Anna O Ermakova^2^*, Azucena Justicia^2,4^, Paul C Fletcher^2,5,6^, Nico Bunzeck^7^, Emrah Düzel^8,9^, Graham K Murray^2,3,5^


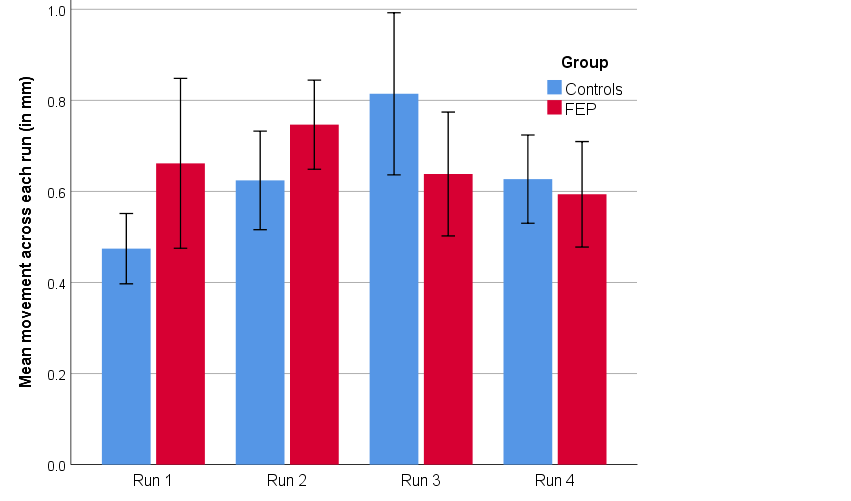


**Supplementary Figure 1.** Mean movement across groups and four scanning runs. Error bars show ±1 SE.


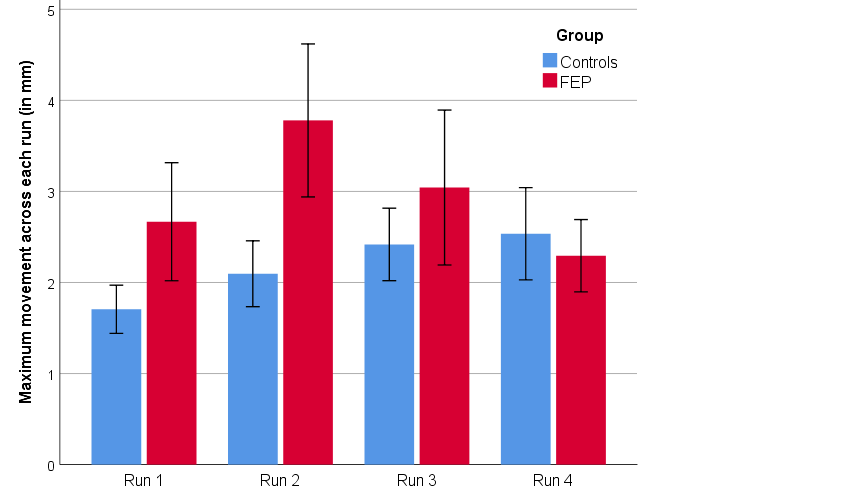


**Supplementary Figure 2.** Maximum movement across groups and four scanning runs. Error bars show ±1 SE.

**
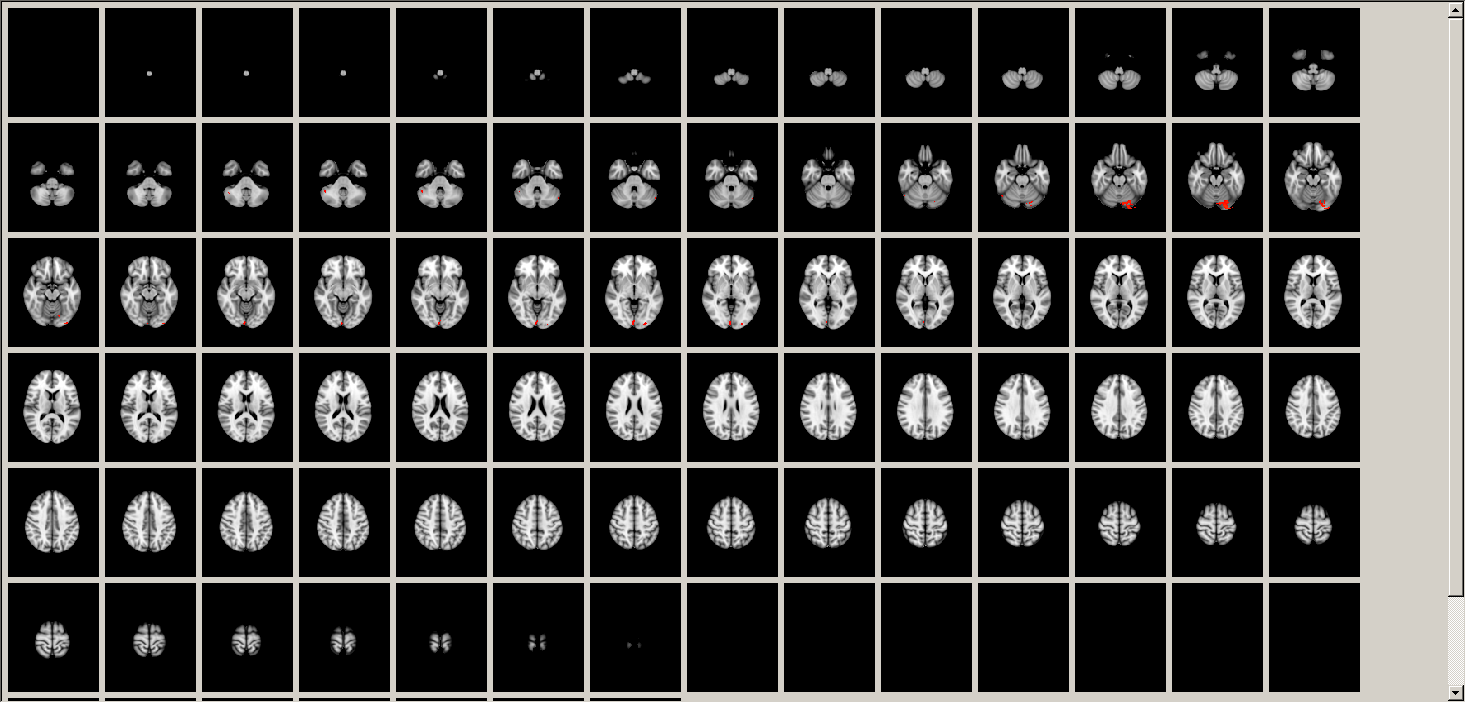
**

**Supplementary Figure 3.** Whole brain group difference analysis results using FSL randomise showing significant group differences (corrected for multiple comparisons) in response to Novelty (novel oddball-neutral oddball), variance smoothing: 3mm, 5000 permutations, and Threshold Free Cluster Enhancement (TFCE).


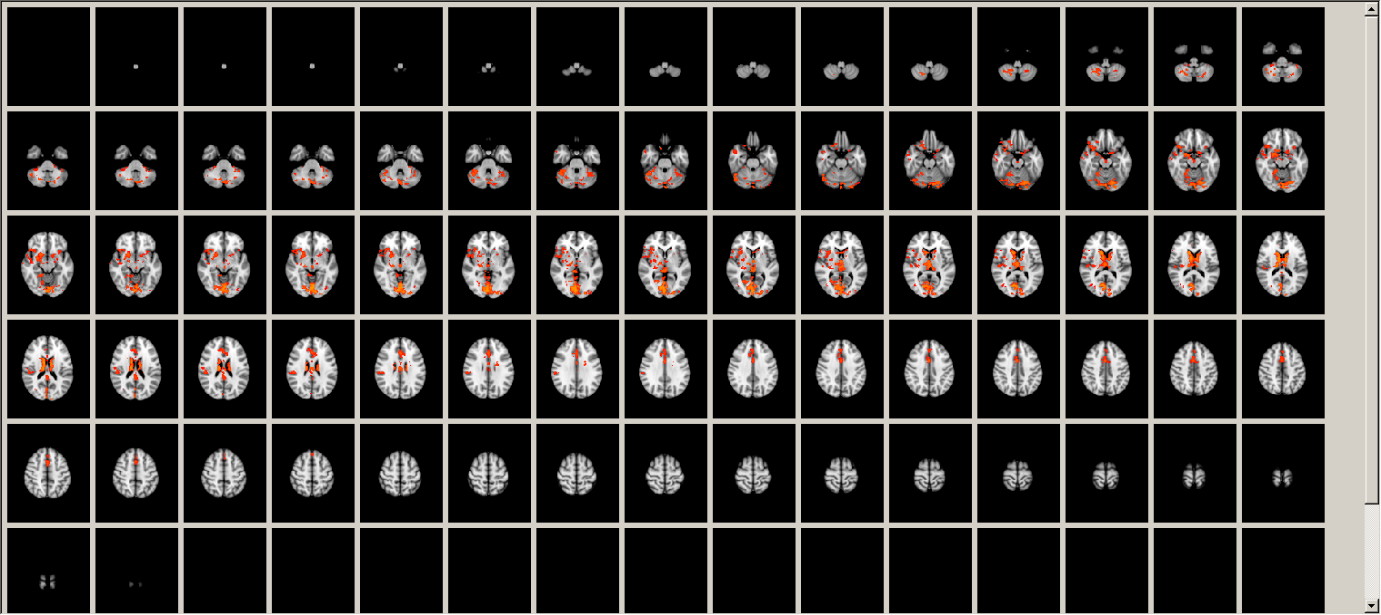
**Supplementary Figure 4.** Whole brain group difference analysis results using FSL randomise showing significant group differences (corrected for multiple comparisons) in response to Negative Emotional Salience (emotional oddball-neutral oddball), variance smoothing: 3mm, 5000 permutations, and Threshold Free Cluster Enhancement (TFCE).


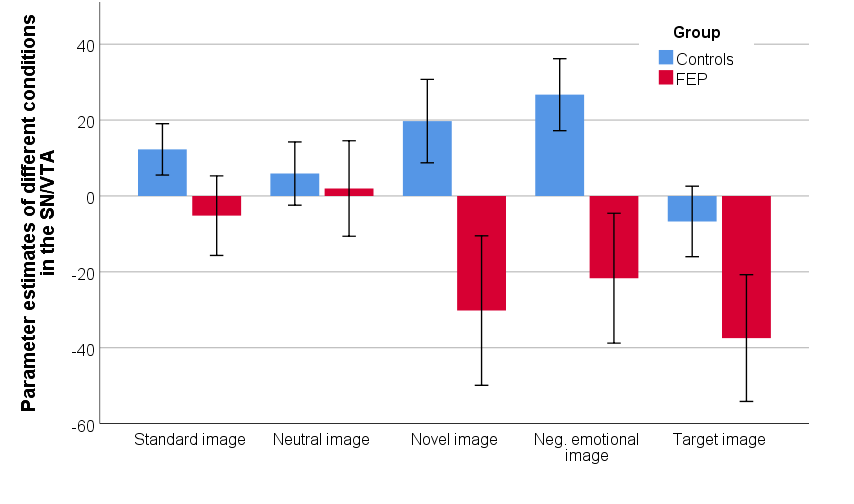


**Supplementary Figure 5.** The bar graph shows activations in the SN/VTA for the FEP patients and control group in response to all different conditions. For parameter estimates extraction the mask for the full ROI was used. The SN/VTA was the primary ROI for all conditions. The differing effect in the group contrast analysis seems to be driven by the inverted pattern of activation in response to novel and negative emotional in the FEPs compared to the control group. Responses to target images show a strong deactivation in patients and significantly reduced effect in controls. Responses to neutral and standard images are similar across groups. Error bars ±1 SE.


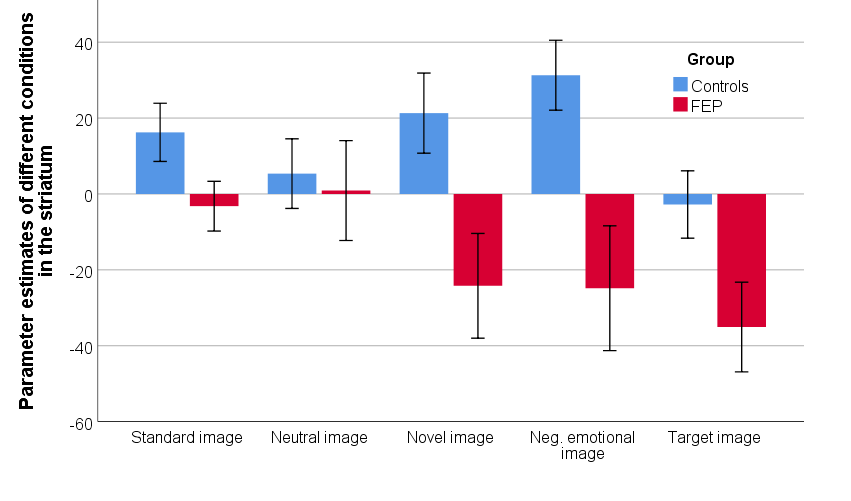


**Supplementary Figure 6.** The bar graph shows activations in the full striatum for the FEP patients and control group in response to all different conditions. For parameter estimates extraction the mask for the full ROI was used. The striatum was the secondary ROI for all conditions. The differing effect in the group analysis seems to be driven by the inverted pattern of activation in response to novel and negative emotional images in the FEPs compared to control group. Responses to target images show a strong deactivation in patients and significantly reduced effect in controls. Responses to neutral and standard images are similar across groups. Error bars ±1 SE.


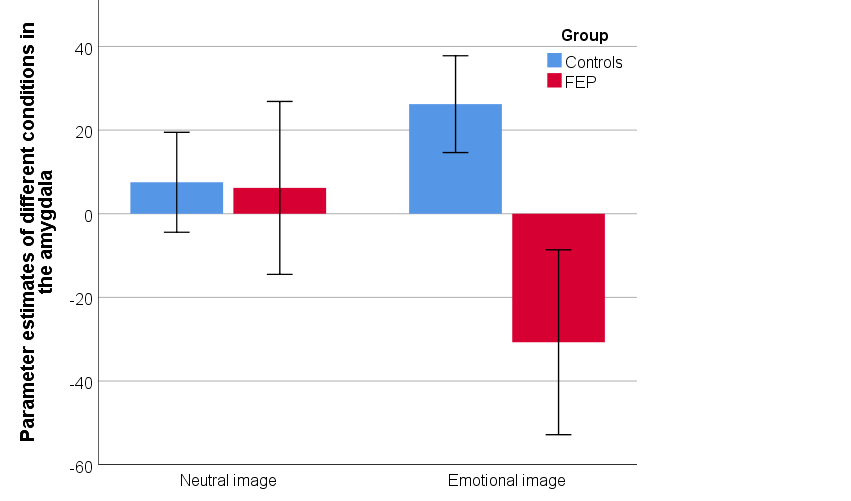


**Supplementary Figure 7.** The bar graph shows activations in the amygdala for the FEP patients and control group in response to neutral and negative emotional images. For parameter estimates extraction the mask for the full ROI was used. The amygdala was the secondary ROI for negative emotional salience. The significant effect in the contrast group analysis seems to be driven by the inverted pattern of activation in response to the negative emotional images in the FEPs compared to control group. Response to neutral images is similar across groups. Error bars ±1 SE.


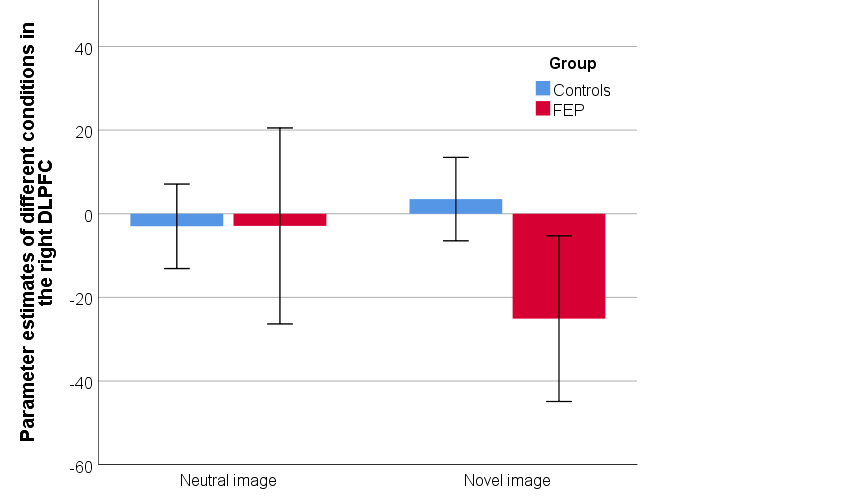


**Supplementary Figure 8.** The bar graph shows activations in the right DLPFC for the FEP patients and control group in response to neutral and novel images. For parameter estimates extraction the mask for the full ROI was used. The right DLPFC was the secondary ROI for novelty salience. We did not find significant difference in the group contrast analysis, which might be influenced by the large standard deviation in the FEP group. Response to novel images appears to show a deactivation in patients compared to controls. Response to neutral images is similar across groups. Error bars ±1 SE.

**
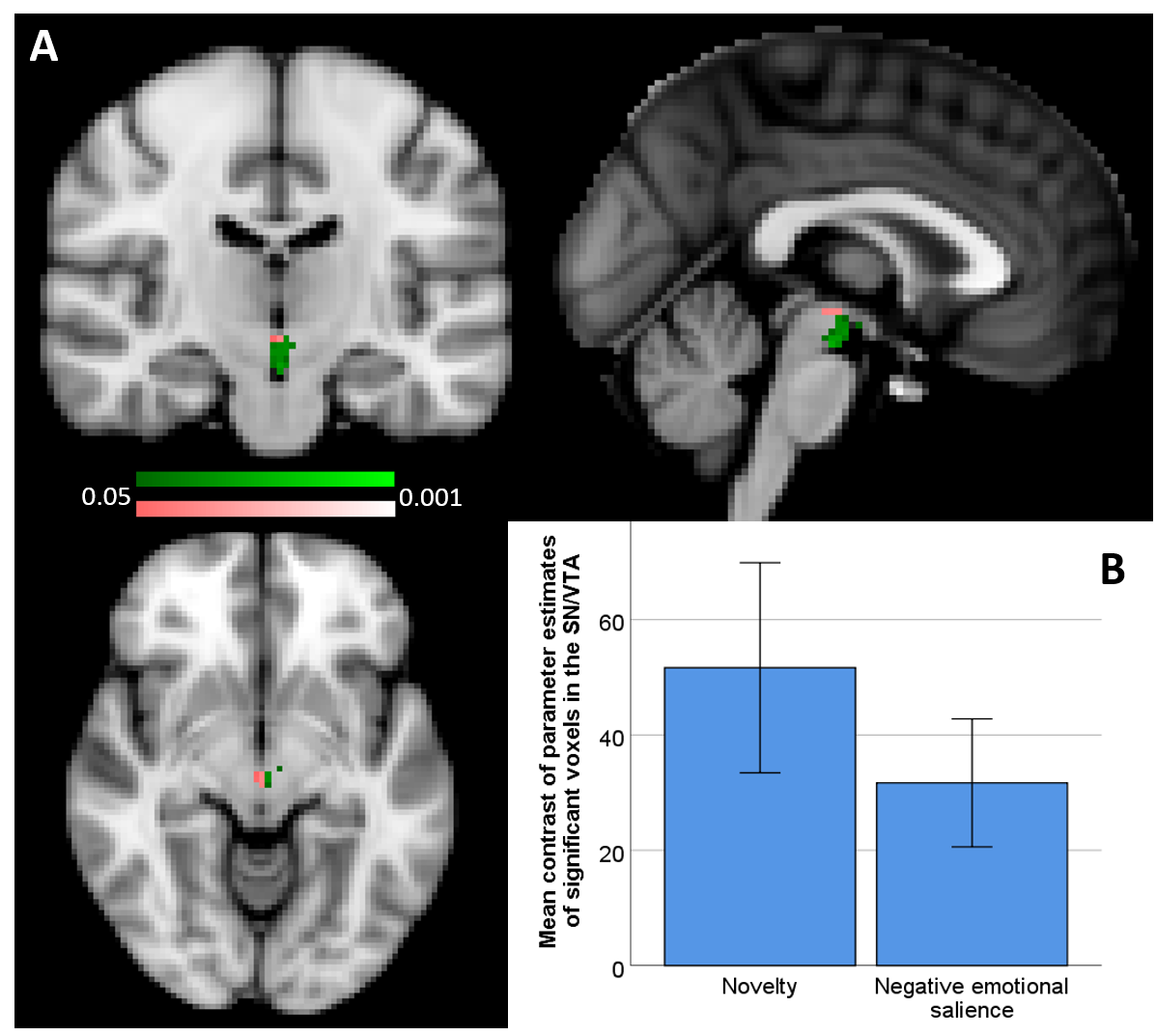
Supplementary Figure 9.** Significant activation in the SN/VTA in response to novelty and negative emotional salience in controls (p<0.05 corrected for multiple comparisons with randomise using TFCE). Panel A shows the location of significantly activated voxels in response to novelty (pink colour coding) and negative emotional salience (green colour coding). Upper left image Y=-18; upper right image x=0; lower left image z=-8. There is no overlap between significantly activated voxels in the two conditions. Panel B shows a bar graph for neural responses to novelty salience and negative emotional salience. Error bars ±1 SE.

**
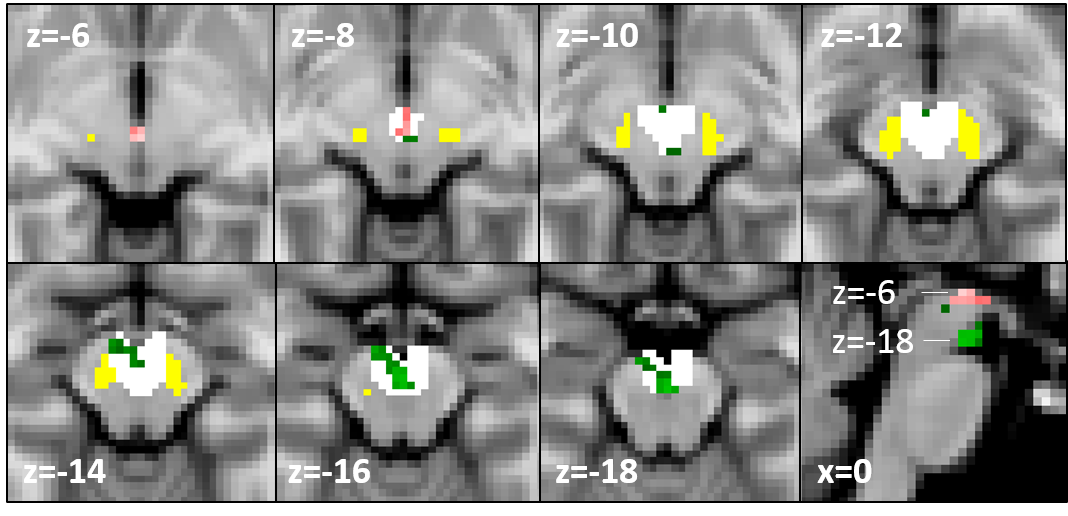
Supplementary Figure 10.** Significant activation in the SN/VTA in response to novelty and negative emotional salience from the group analysis (p<0.05 corrected for multiple comparisons with randomise using TFCE). Each panel shows the location of significantly activated voxels in response to novelty (pink colour coding) and negative emotional salience (green colour coding) at a different level, z=-6 to z=-18, overlaid on the masks used for the analysis, white mask shows VTA and yellow mask shows SN. There is no overlap between significantly activated voxels in the two conditions.


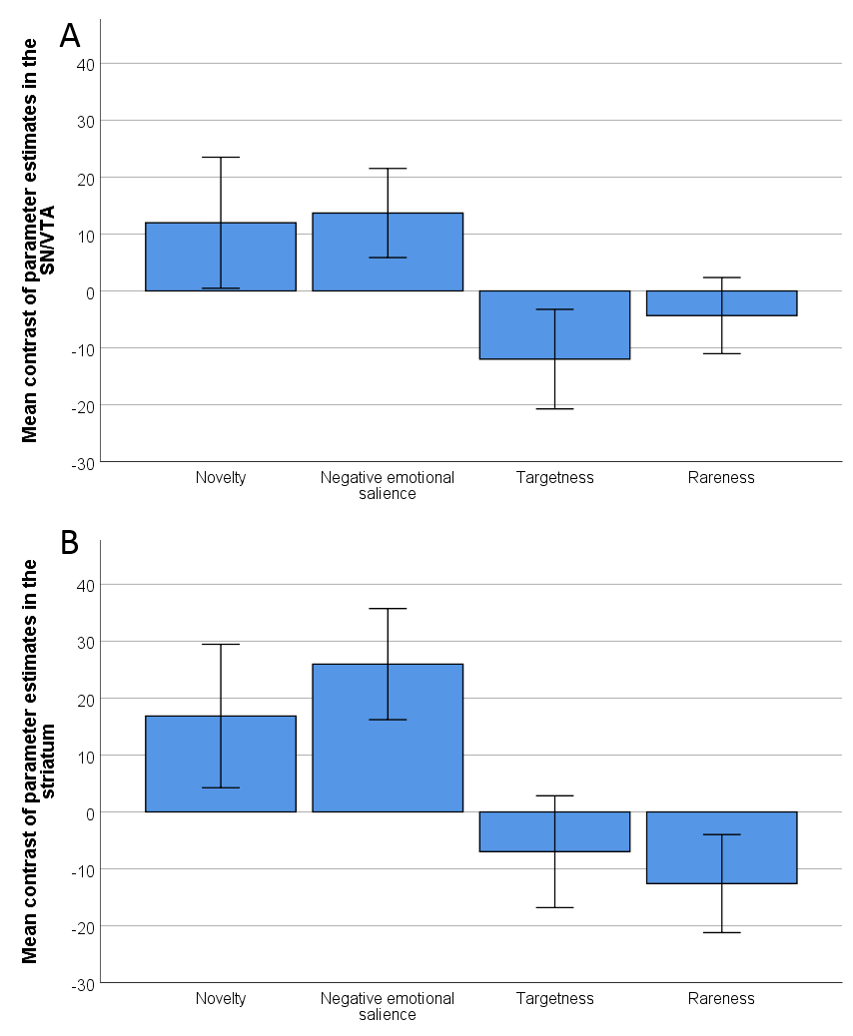


**Supplementary Figure 11.** The bar graphs show contrast values averaged over all ROI voxels in the SN/VTA (A) and the striatum (B) for controls in response to four different types of salience. A) neural responses to novelty salience and negative emotional salience are significantly enhanced compared to targetness (novelty>targetness: p=0.013; negative emotional salience>targetness: p=0.008). B) neural responses to negative emotional salience is significantly enhanced compared to targetness (p=0.011). Bonferroni corrected pairwise comparisons. Error bars ±1 SE.

| **Supplementary Table 1**: Means and standard deviations of the missed buttons and reaction times. | | | | |  |
| --- | --- | --- | --- | --- | --- |
|  | Controls | | FEP | | |
|  | Mean | SD | Mean | SD | |
| Missed button presses | 0.86 | 1.2 | 1.11 | 1.1 | |
| Reaction times (s) | 0.55 | 0.1 | 0.54 | 0.2 | |
| FEP, first episode psychosis patients. SD, standard deviation. Mean values do not include those rejected runs. | | | | |  |

| **Supplementary Table 2.** Correlations between regional activations separated by group. | | | | | | | | | | | | | |
| --- | --- | --- | --- | --- | --- | --- | --- | --- | --- | --- | --- | --- | --- |
|  |  | SN/VTA to Novelty | | SN/VTA to Neg. Emotional Salience | | SN/VTA to Targetness | | Striatum to Novelty | | Striatum to Neg. Emotional Salience | | Amygdala to Neg. Emotional Salience | |
|  |  | Control | FEP | Control | FEP | Control | FEP | Control | FEP | Control | FEP | Control | FEP |
| SN/VTA to Novelty | rho | 1.000 | 1.000 | 0.165 | 0.363 | **.436^**^** | 0.500 | **.421^*^** | **.709^**^** | 0.216 | 0.077 | **.372^*^** | 0.231 |
|  | P value |  |  | 0.350 | 0.223 | **0.010** | 0.082 | **0.013** | **0.007** | 0.220 | 0.803 | **0.030** | 0.448 |
|  | N |  |  | 34 | 13 | **34** | 13 | **34** | **13** | 34 | 13 | **34** | 13 |
| SN/VTA to Neg. Emotional Salience | Rho | 0.165 | 0.363 | 1.000 | 1.000 | 0.321 | 0.522 | **.449^**^** | 0.099 | **.697^**^** | 0.368 | **.712^**^** | **.659^*^** |
|  | P value | 0.350 | 0.223 |  |  | 0.064 | 0.067 | **0.008** | 0.748 | **0.000** | 0.216 | **0.000** | **0.014** |
|  | N | 34 | 13 |  |  | 34 | 13 | **34** | 13 | **34** | 13 | **34** | **13** |
| SN/VTA to Targetness | rho | **.436^**^** | 0.500 | 0.321 | 0.522 | 1.000 | 1.000 | 0.152 | **.654^*^** | 0.198 | 0.302 | 0.198 | 0.374 |
|  | P value | **0.010** | 0.082 | 0.064 | 0.067 |  |  | 0.392 | **0.015** | 0.263 | 0.316 | 0.260 | 0.209 |
|  | N | **34** | 13 | 34 | 13 |  |  | 34 | **13** | 34 | 13 | 34 | 13 |
| Striatum to Novelty | rho | **.421^*^** | **.709^**^** | **.449^**^** | 0.099 | 0.152 | **.654^*^** | 1.000 | 1.000 | **.575^**^** | 0.308 | **.562^**^** | 0.291 |
|  | P value | **0.013** | **0.007** | **0.008** | 0.748 | 0.392 | **0.015** |  |  | **0.000** | 0.306 | **0.001** | 0.334 |
|  | N | **34** | **13** | **34** | 13 | 34 | **13** |  |  | **34** | 13 | **34** | 13 |
| Striatum to Neg. Emotional Salience | rho | 0.216 | 0.077 | **.697^**^** | 0.368 | 0.198 | 0.302 | **.575^**^** | 0.308 | 1.000 | 1.000 | **.797^**^** | **.753^**^** |
|  | P value | 0.220 | 0.803 | **0.000** | 0.216 | 0.263 | 0.316 | **0.000** | 0.306 |  |  | **0.000** | **0.003** |
|  | N | 34 | 13 | **34** | 13 | 34 | 13 | **34** | 13 |  |  | **34** | **13** |
| Amygdala to Neg. Emotional Salience | Rho | **.372^*^** | 0.231 | **.712^**^** | **.659^*^** | 0.198 | 0.374 | **.562^**^** | 0.291 | **.797^**^** | **.753^**^** | 1.000 | 1.000 |
|  | P value | **0.030** | 0.448 | **0.000** | **0.014** | 0.260 | 0.209 | **0.001** | 0.334 | **0.000** | **0.003** |  |  |
|  | N | **34** | 13 | **34** | **13** | 34 | 13 | **34** | 13 | **34** | **13** |  |  |
| **. Correlation is significant at the 0.01 level (2-tailed). | | | | | | | | | | | | | |
| *. Correlation is significant at the 0.05 level (2-tailed). | | | | | | | | | | | | | |

| **Supplementary Table 3:** fMRI Activations from FSL randomise whole brain analysis for controls only. | | | | | | | |
| --- | --- | --- | --- | --- | --- | --- | --- |
| Anatomical structure | Hemisphere | Cluster size (voxel) | P value  (TFCE) | T value | Peak MNI-coordinates | | |
|  |  |  |  |  | X | y | z |
|  |  |  |  |  |  |  |  |
| Novel – Neutral Oddball (Novelty) | | | | | | | |
| n.s. |  |  |  |  |  |  |  |
| Emotional – Neutral Oddball (Negative emotional salience) | | | | | | | |
| Frontal Orbital Cortex | R | 16241 | **0.017** | 4.37 | 44 | 22 | -12 |
| Lateral Occipital Cortex | R | 22 | **0.049** | 3.12 | 42 | -70 | 12 |
| Supra- and intrcalcarine Cortex | R/L | 16 | **0.048** | 3.59 | 0 | -76 | 14 |
| Lingual Gyrus, intracalcalrine cortex | R/L | 13 | **0.049** | 3.44 | 2 | -84 | 0 |
| Intracalcarine Cortex | R | 11 | **0.049** | 3.47 | 8 | -72 | 16 |
| Lateral Occipital Cortex | R | 10 | **0.049** | 3.14 | 36 | -74 | 28 |
| Insula | L | 9 | **0.048** | 3.43 | -38 | -20 | -2 |
| Target – Neutral Oddball (Targetness) | | | | | | | |
| n.s. |  |  |  |  |  |  |  |
| Neutral – Standard Oddball (Rareness) | | | | | | | |
| n.s. |  |  |  |  |  |  |  |
| Summary of fMRI results for significant clusters at the whole-brain level, using FSL randomise, variance smoothing: 3mm, 5000 permutations, and Threshold Free Cluster Enhancement (TFCE). **Bold**: significant differences. N.s., not significant. | | | | | | | |

| **Supplementary Table 4:** fMRI Activations from ROI analysis for controls only. | | | | | | | |
| --- | --- | --- | --- | --- | --- | --- | --- |
| Anatomical structure | Hemisphere | Cluster size (voxel) | P value  (TFCE) | T value | Peak MNI-coordinates | | |
|  |  |  |  |  | x | y | z |
|  |  |  |  |  |  |  |  |
| Novel – Neutral Oddball (Novelty) | | | | | | | |
| SN/VTA |  | 5 | **0.038** | 3.3 | 0 | -20 | -8 |
| Striatum and DLPFC | R | 45 | 0.056 | 3.89 | 8 | -2 | 14 |
| Marginally significant clusters only within the striatum | L | 26 | 0.082 | 3.25 | -12 | -16 | 18 |
| Emotional – Neutral Oddball (Negative emotional salience) | | | | | | | |
| SN/VTA |  | 55 | **0.027** | 3.43 | 0 | -20 | -18 |
| Striatum and Amygdala | L | 2960 | **0.007** | 3.89 | -8 | 4 | -2 |
| Target – Neutral Oddball (Targetness) | | | | | | | |
| SN/VTA |  |  | n.s. |  |  |  |  |
| Striatum |  |  | n.s. |  |  |  |  |
| Neutral – Standard Oddball (Rareness) | | | | | | | |
| SN/VTA |  |  | n.s. |  |  |  |  |
| Striatum |  |  | n.s. |  |  |  |  |
| Summary of fMRI results for region of interest (ROI), using FSL randomise, variance smoothing: 3mm, 5000 permutations, and Threshold Free Cluster Enhancement (TFCE). **Bold**: significant differences. n.s., not significant. | | | | | | | |
